# Overexpression of SIRT3 disrupts mitochondrial proteostasis and cell cycle progression

**DOI:** 10.1007/s13238-016-0251-z

**Published:** 2016-02-18

**Authors:** Xiaofei Wang, Haiping Tang, Yuling Chen, Binghuan Chi, Shiyu Wang, Yang Lv, Di Wu, Renshan Ge, Haiteng Deng

**Affiliations:** MOE Key Laboratory of Bioinformatics, School of Life Sciences, Tsinghua University, Beijing, 100084 China; Wenzhou Medical College, Wenzhou, 325035 China; Center of Nephrology, The General Hospital of the PLA, Beijing, 100853 China

**Dear Editor,**

As a mitochondrial deacetylase, SIRT3 deacetylates many enzymes involved in central metabolism and maintains mitochondrial proteostasis (Verdin et al., 2010; Papa and Germain, 2014). Substrates of SIRT3 include components of the respiratory complexes, proteins involved in fatty acid oxidation and TCA cycle (Yu et al., 2012). SIRT3 activates MnSOD to maintain reactive oxygen species (ROS) homeostasis and a loss of SIRT3 contributes to the age-associated diseases (McDonnell et al., 2015; Qiu et al., 2010). SIRT3 plays dual roles functioning as a tumor suppressor or a promoter in tumorigenesis and progression (Alhazzazi et al., 2011). On one hand, SIRT3 regulates the cellular ROS level and maintains genomic stability, and mediates metabolic reprogramming to prevent tumorigenesis (Finley and Haigis, 2012). As a result, the low expression of SIRT3 has been found in breast cancer, glioblastoma, colon cancer, osteosarcoma, prostate, and ovarian cancers (Kim et al., 2010; Finley and Haigis, 2012). On the other hand, SIRT3 is a prosurvival factor that modulates p53 activities and is upregulated in oral cancer, the node-positive breast cancer, and bladder cancer (Ashraf et al., 2006; Alhazzazi et al., 2011). These results suggest that SIRT3 possesses the tumor-type dependent function and its precise role needs to be elucidated in the context of a specific cancer. Clear cell renal cell carcinoma (ccRCC) is the most common histological subtype of renal cancer (Cohen and McGovern, 2005). The aims of the present study were to examine the expression of SIRT3 in ccRCC and to characterize effects of SIRT3 on tumorigenesis and progression using 293T human embryonic kidney cells as the model system that has cancer stem cell-like features (Debeb et al., 2010).

Equal amounts of proteins extracted from 18 paired ccRCC lesions and associated pericarcinous tissue samples were analyzed by Western blotting and the representative Western blot images of eight paired samples were shown in Fig. 1A, indicating that the expression levels of SIRT3 were lower in ccRCC than those in normal tissues. The gray scale analysis of the Western blot data for all eighteen paired samples showed that the SIRT3 expression was statistically down-regulated in ccRCC tissues (Fig. 1B), suggesting that the low expression of SIRT3 is important for ccRCC progression.

**Figure 1:**
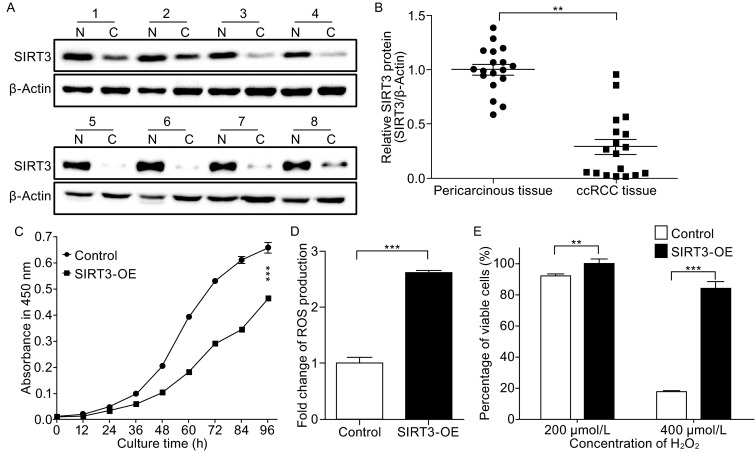
**Downregulation of SIRT3 in ccRCC compared to associated pericarcinous tissues and characterization of SIRT3 overexpression cells**. (A) Representative Western blot images of the expression levels of SIRT3 of eight paired samples, N (pericarcinous tissue), C (ccRCC tissue). (B) The gray scale analysis of SIRT3 presented in (A). (C) Growth curve of SIRT3-OE and the control cells. (D) Graphical representation of ROS levels of SIRT3-OE cells compared to the control cells. and (E) Survival rate of SIRT3-OE cells and the control cells treated with different concentration of H_2_O_2_ for 12 h. Data were analyzed using student’s *t*-test. *P < 0.05, **P < 0.01 and ***P < 0.001. *P < 0.05 is considered statistically significant. Error bars represent ±SEM

To understand the role of SIRT3 in tumorigenesis and progression of ccRCC, stable cells overexpressing SIRT3 were established in 293T cells. The overexpression of SIRT3 in 293T cells (SIRT3-OE) was examined by Western blotting (Fig. S1), confirming that the expression level of SIRT3 in SIRT3-OE cells was four fold higher than that in control cells. The SIRT3 overexpression in 293T led to a decrease in proliferation rates (Fig. 1C). The ROS level in SIRT3-OE cells is two and half fold higher than that in the control cells as detected using the CellROX^®^ Deep Red kit (Fig. 1D). To determine the susceptibility of SIRT3-OE cells to oxidative stress, cells were treated with various concentrations of hydrogen peroxide for 12 h. The cell viability was measured using CCK-8 assay. The effects of hydrogen peroxide were represented as the percentage of viable cells after 12 h treatment (Fig. 1E). When cells were treated with 400 µmol/L H_2_O_2_ for 12 h, the percentages of viable cells were 20% and 90% for the control and SIRT3-OE cells, respectively (Fig. 1E). This declares that SIRT3-OE cells are more resistant to H_2_O_2_ treatment.

Next, proteomic analysis was carried out on SIRT3-OE and control cells in biological replicates. Equal amounts of proteins from SIRT3-OE and the control cells were in-solution digested and labeled with TMT reagents. The generated tryptic peptides were fractionated using off-line HPLC and each fraction was further analyzed by nano-LC-MS/MS. Differentially expressed proteins were identified and quantified using the TMT-based quantification. We identified 7536 proteins in two biological replicates and the false-positive rate was estimated to be less than 1%. Based on the average reporter ion ratios (>1.5 or <0.67), 188 proteins were found to be differentially expressed between SIRT3-OE and control cells, in which 93 proteins were down-regulated and 95 were up-regulated (Tables S1 and S2). To understand the biological relevance of the differentially expressed proteins, the Gene Ontology (GO) was used to cluster differentially expressed proteins according to their associated biological processes. The annotations of gene lists are summarized via a pie plot based on the functional classification from Panther as shown in Fig. 2A. One hundred and eighty eight differentially expressed proteins participated in a variety of cellular processes including metabolic process, cellular process, and cellular component organization process. The primary metabolic process shows the dominant difference between SIRT3-OE and the control cells. About 25% of the down-regulated proteins are classified as mitochondrial proteins, indicating that SIRT3 overexpression has a great impact on the mitochondrial protein expressions. Five subunits of respiratory complex IV were down-regulated in SIRT3-OE cells including COX7C, COX6A1, COX7A2, COA7, and COA5 (Fig. 2B), suggesting that the SIRT3 overexpression disrupted the integrity of the respiratory complexes. We also identified that three proteins in the fatty acid β-oxidation pathway were downregulated in SIRT3-OE cells including enoyl-CoA hydratase, very long-chain specific acyl-CoA dehydrogenase and hydroxyacyl-coenzyme A dehydrogenase. More importantly, proteomic analysis showed that nine subunits of mitochondrial ribosomes were downregulated in SIRT3-OE cells (Fig. 2B). All these results indicated that SIRT3-overexpression disrupted mitochondrial proteostasis and contributed to mitochondrial dysfunction.

**Figure 2:**
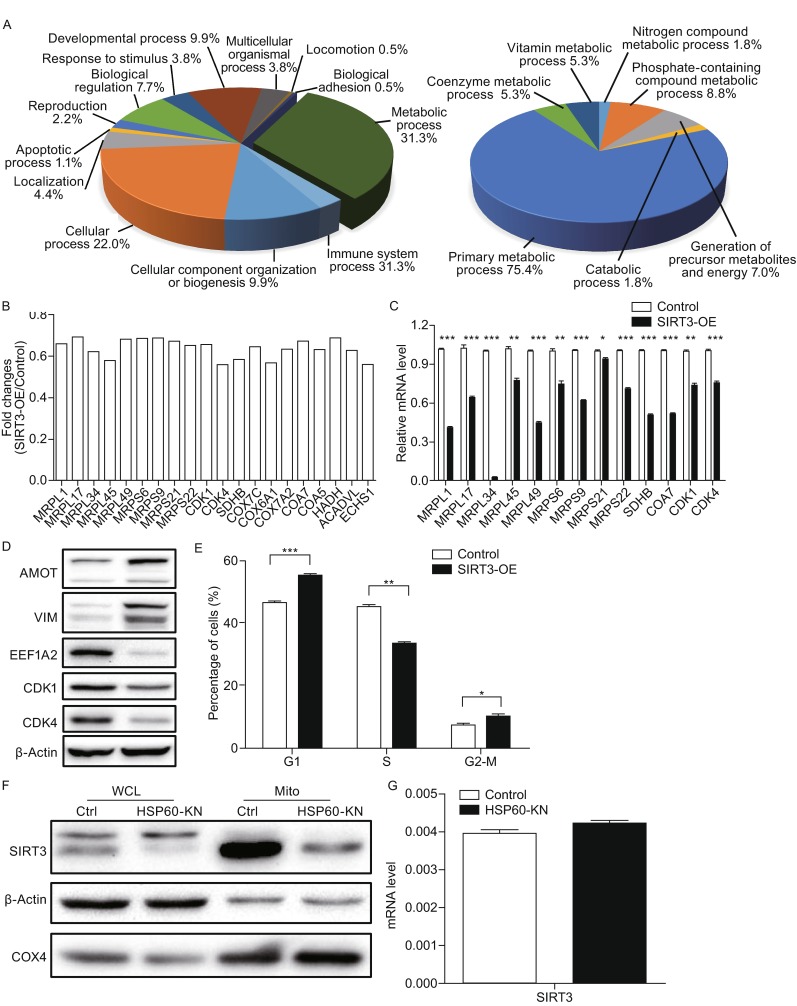
**Proteomic, qPCR and Western blot analysis of differentially expressed proteins between SIRT3-OE and control cells**. (A) GO analysis of the differentially expressed proteins in SIRT3-OE cells compared to the control cells with PANTHER (http://www.pantherdb.org). (B) Graphical representation of TMT ratios for proteins in SIRT3-OE cells compared to control cells. (C) qPCR analysis of mRNA expressions of selected mitochondrial ribosomal genes and other selected genes from SIRT3-OE cells and control cells. (D) Western blot analysis of selected proteins from SIRT3-OE and control cells. (E) Cell cycle analysis of SIRT3-OE cells and the control cells. (F) Western blotting images showing expression level of SIRT3 in HSP60-KN cells compared to the control cells, WCL (whole cell lysates), Mito (mitochondria); and (G) mRNA expression level of SIRT3 in HSP60-KN cells and control cells. Data were analyzed using student’s *t*-test. *P < 0.05, **P < 0.01 and ***P < 0.001. Error bars represent ±SEM

qPCR analysis was conducted and showed that the mRNA expression levels of these nine mitochondrial ribosomal genes were lower in SIRT3-OE cells than those in the control cells (Fig. 2C). qPCR analysis also confirmed the downregulation of other genes including SDHB, COA7, CDK1, and CDK4 (Fig. 2C). Additionally, Western blotting was employed to examine expressions of the selected proteins and showed that the expression levels of EEF1A2, CDK1, and CDK4 were down-regulated whereas those of vimentin and angiomotin were upregulated in SIRT3-OE cells (Fig. 2D), consistent with the proteomic results displayed in Tables S1 and S2. The above results also showed that SIRT3-OE cells grew slower than the control cells (Fig. 1C), indicating that cell cycle progression varies between those two cells. Indeed, the cell cycle analysis of SIRT3-OE and control cells showed that SIRT3-OE cells had the higher G_1_-phase accumulation (Fig. 2E), in consistent with the down-regulation of CDK1 and CDK4 in SIRT3-OE cells.

SIRT3 is known to play a crucial role in maintenance of mitochondrial proteostasis. Proteomic analysis showed that multiple subunits of respiratory complex IV were down-regulated in SIRT3-OE cells, which compromised the integrity and assembly of respiratory complexes, leading to over-production of ROS (Fig. 1D). Excessive ROS can negatively regulate cell growth and may activate the Nrf2/Keap1 pathway that protects SIRT3-OE cells from oxidative stress (Fig. 1E). An alternative explanation for the high resistance to oxidative stress exhibited in SIRT3-OE cells is that deacetylation of antioxidant proteins by SIRT3 enhances their enzymatic activities, which needs to be confirmed in the future study. Three key proteins in fatty acid β-oxidation pathway were also down-regulated in SIRT3-OE cells (Table S2). Growth and proliferation of tumor cells require fatty acids for synthesis of membranes and signaling molecules. Disruption of fatty acid oxidation pathway causes a decrease in both acetyl-CoA production that is essential for the *de novo* lipid synthesis and NADH and FADH_2_ generation that are important for ATP and citrate production (Carracedo et al., 2013). Moreover, SIRT3-meidated disruption of fatty acid β-oxidation can lead to the accumulation of fatty acids to induce lipotoxicity. Cyclin-dependent kinase 1 (CDK1) and cyclin-dependent kinase 4 (CDK4) were found to be downregulated in SIRT3-OE cells as confirmed by qPCR and Western blotting. CDK4 is associated with D-type cyclins to promote cell-cycle entry and progression through G_1_ by inactivating the retinoblastoma protein Rb (Sherr and Roberts, 1999). SIRT3-induced CDK4 downregulation causes the prolonged G_1_ cell cycle arrest that contributes to the slower growth of SIRT3-OE cells as compared to the control cells.

To further identify factors that regulate SIRT3 stability, we isolated the SIRT3 complexes from SIRT3-OE cells. Protein components of the SIRT3 complexes were separated on a 1D SDS-PAGE gel and changes in band intensities were identified between SIRT3-OE and the control cells (Fig. S2). These bands were excised, digested by trypsin and analyzed by LC-MS/MS, resulting in the identification of HSP60 as the major binding partner of SIRT3. Similarly, immunoprecipitation of HSP60 from 293T cells was carried out and showed that SIRT3 bound to HSP60, suggesting that SIRT3 directly interacts with HSP60. In order to confirm that HSP60 regulates SIRT3 stability, we established the HSP60 knockdown cells. Western blotting showed that SIRT3 was down regulated in HSP60 knockdown cells as compared to the control cells (Fig. 2F). On the other hand, qPCR analysis revealed that the SIRT3 mRNA level was unchanged between these two cells, suggesting that HSP60 regulated SIRT3 stability (Fig. 2G). This is consistent with an early study showing that two murine SIRT3 isoforms interacted with HSP60 (Yang et al., 2011). HSP60 is the major mitochondrial chaperone and is essential in maintenance of mitochondrial proteostasis. More work is needed to examine the molecular mechanisms of SIRT3 degradation in HSP60-knockdown cells. Nevertheless, our results propose that HSP60 is important in the maintenance of SIRT3 protein stability.

Taken together, we demonstrate that SIRT3 overexpression disrupts mitochondrial proteostasis that causes overproduction of ROS and the cell cycle arrest to suppress cell proliferation, proposing that the low level expression of SIRT3 is important for tumorigenesis and progression in ccRCC. Our data also show that HSP60 mediates the stability of SIRT3 and proteomics is a powerful approach to decipher the complex cellular processes.

## Electronic supplementary material

Below is the link to the electronic supplementary material.

Supplementary material 1 (PDF 386 kb)
