# Supplementary material for: Overexpression of SIRT3 disrupts mitochondrial proteostasis and cell cycle progression
Source: Protein Cell. 2016 Feb 18;7(4):295–9. doi: 10.1007/s13238-016-0251-z (PMC4818846; doi:10.1007/s13238-016-0251-z)
Supplement: Supplementary file 1 — Supplementary material 1 (PDF 386 kb) [file 13238_2016_251_MOESM1_ESM.pdf]

MATERIALS AND METHODS

Chemicals and Reagents

Dulbecco's Modified Eagle Medium(DMEM), fetal bovine serum, and penicillin-streptomycin were purchased from Wisent (Saint-Jean-Baptiste, CA). The 293T cell line was obtained from the Tumor Cell Bank of the Chinese Academy of Medical Sciences (Beijing, China). Dithiothreitol (DTT) and BCA protein assay kit were purchased from Solarbio (Beijing, China). Sequencing grade modified trypsin was purchased from Promega (Fitchburg, WI). Mass spectrometry grade acetonitrile was purchased from Thermo (Waltham, MA). The TMT labeling kit was purchased from Thermo-Pierce Biotechnology (Rock-ford, IL). Cell counting kit-8 was purchased from Dojindo (Kumamoto, Janpan). H_2O_2 was purchased from Aladdin (Shanghai, China). The Total RNA Isolation System and Reverse Transcription kits were purchased from TIANGEN (Beijing, China). AceQ qPCR SYBR Green Master Mix was purchased from Vazyme (Nanjing, China). anti-SIRT3 monoclonal antibody were obtained from Cell Signaling Technology (Danvers, MA).

Clinical human ccRCC specimens

18 pairs of ccRCC and pericarcinous tissue samples were collected after written informedconsent from patients with ccRCC undergoing surgery at the Department of Urological Surgery of the General Hospital of PLA during 2013–2014 (Beijing, China). The group was composed of 14 men and 4 women with a mean age of 50.3 years (range 29–66 years) at the time of operation. The study was approved by the Scientific and

Ethnic Committee of the General Hospital of PLA, Kidney cancer tissues were surgically removed from ccRCC patient while samples of pericarcinous tissues were obtained from the distal edge of the resection at least 6 cm from the tumor tissue. Part of the specimen was directly snap-frozen in liquid nitrogen, and stored at -80°C before extraction of total proteins. The other part of specimen was fixed in buffered formalin for 48 h, embedded in paraffin, and sectioned into 5 LM slides for immunohistochemistry (IHC) examination. Total proteins were extracted from paired tissues using 8M Urea in PBS (pH 7.4) followed by western blotting.

Cell Culture and Establishment of stable SIRT3 overexpression cell line

The human embryonic kidney cell line 293T cells were maintained in DMEM media (Wisent, Montreal, QC) supplemented with 10% fetal bovine serum (Wisent, Montreal, QC) and 1%penicillin/streptomycin (Wisent, Montreal, QC) at 37℃ in a humidified incubator with 5% CO_2.

Lentiviral expression vector pLVX-IRES-ZsGreen1 with reporter gene of GFP and the packaging vectors were gifts from Dr. Jun Xu (Tongji University, Shanghai, China). The human SIRT3 cDNA was synthesized from the total RNA of 293T cell line. A FLAG-tag sequence was added onto the C-terminal of SIRT3 DNA. The recombinant human SIRT3 DNA was cloned into pLVX-IRES-ZsGreen1 to create the pLVX-SIRT3-IRES-ZsGreen1 vector. Then, we co-transfected pLVX-SIRT3-IRES-ZsGreen1 or pLVX-IRES-ZsGreen1, respectively, with packing vectors into 293T cells when they reached 80-90% confluence. The cell culture supernatant was then collected

after 48 h and was concentrated with PEG6000. The precipitated lentiviral particles were resuspended in PBS.

293T cells were cultured in a 12-well plate and lentiviral particles with 5 μg ml^{-1} polybrene were added into 293T cells when they reached 30–40% confluence. After 72 h, a large population of cells expressed GFP and emitted green fluorescence. Cells were sorted by a flow cytometer to generate the monoclonal stable cell line. The clone with intense and uniform GFP expression was selected and used in the present study. The expression of SIRT3 was examined by western blotting and qPCR.

Cell proliferation assay with CCK-8

Cells were seeded in 96-well plates with 2500 cells/well. CCK-8 reagents were added into wells after cells grew for 0, 12 24, 36, 48, 72, 84, 96 h respectively. The CCK8 reagent was added to medium according to the manufacturer's instructions (Dojindo Laboratories, Kumamoto, Japan) and incubated at 37 ℃ for 2 h. Optical density (OD) was measured at 450 nm with a microplate reader (Bio-Rad, Hercules).

Survival rate of SIRT3 overexpression cells treated with hydrogen peroxide

Effects of hydrogen peroxide on control and SIRT3 overexpression cells were analyzed with the CCK-8 kit. Briefly, Cells were treated with hydrogen peroxide (200 and 400μM) in triplicates for 12 hours. The CCK-8 reagent was added to treated cells and incubated at 37 ℃ for 2 h. Absorbance at 450 nm was measured 2 h after CCK-8 addition.

Detection of cellular reactive oxygen species (ROS)

The ROS in SIRT3 overexpression cells was detected using CellROX® Deep Red Reagents (Invitrogen, Grand Island, NY) according to the manufacturer's instructions. Briefly, adding the probe to the complete medium with 5 μM CellROX® Deep Red Reagent and incubating the cells at 37 ℃ for 30 minutes. The cells were then washed with HBSS and analyzed on a BD FACSAriaII Flow Cytometer (BD Biosciences, San Jose, CA).

Cell Cycle Analysis

The Cells were seeded in 6-well plates with about $1x10^6$ cells/well and allowed to grow for 24 hours. Then, cells were harvested, washed with cold PBS, and fixed overnight in pre-cooled 70% ethanol at 4 ℃. After that, cells were washed with cold PBS and re-suspended in a PBS solution containing 25 mg/ml RNase A at 37 ℃ for 30 minutes. Then cells were incubated with 10mg/ml propidium iodide (PI) solution in dark at 4 ℃ for 1 hour before being transferred to FACS tubes for flow cytometry analysis. DNA content was analyzed using BD FACSCalibur (Becton Dickinson, Franklin Lakes, NJ) and data were acquired and analyzed using the CELLQuest software from Becton Dickinson.

Sample Preparation and Quantitative Proteomic Analysis

Proteomic analysis was carried out in biological duplicates. Cells were washed twice with PBS, and lysed with 8 M Urea in PBS. Protein concentrations were measured by the BCA method. Equal amount of proteins from 293T control and 293T SIRT3

overexpression cells (100 μg) were reduced with 5 mM dithiothreitol (DTT) and alkylated with 12.5 mM iodoacetamide (IAA). Samples were diluted with PBS to 1.5 M Urea followed by digestion with trypsin of a 1:100 protease/protein ratio at 37 ℃ overnight. Samples were desalted using Sep-Pak C18 cartridges. Purified peptides were labeled with TMT reagents (Thermo, Pierce Biotechnology, Rockford, IL) according to the manufacture's instruction. Briefly, the TMT labeling reagents was dissolved in anhydrous acetonitrile and added to each digested products, then the reaction was incubated at room temperature for 1 hour, and quenched by 5% hydroxylamine for 15 min. The TMT labeled peptides were mixed and desalted by desalted by Sep-Pak C18 cartridges. The peptides were fractionated by a UPLC3000 system (Dionex, CA) with a XBridge TMBEH300 C18 column (Waters, MA). Mobile phase A is H_2O with ammonium hydroxide, pH 10; and mobile phase B is acetonitrile in ammonium hydroxide pH 10. Peptides were separated with the following gradients: 8% to 18% phase B, 30 min; 18% to 32% phase B, 22 min;48 fractions were collected, dried by a speedvac, combined into 12 fractions, and redissolved in 0.1% formic acid for the following nano-LC-MS/MS analysis.

For quantitative proteomics analysis, the peptides were separated by a C_{18} column (75 μm inner-diameter, 150 mm length; Upchurch, Oak Harbor, USA) at a flow rate of 250 nL/min. Mobile phase A consisted of 0.1% formic acid, and mobile phase B consisted of 100% acetonitrile and 0.1% formic acid. The Q-Exactive mass spectrometer was operated in the data-dependent acquisition mode using Xcalibur 3.0 software: there was a single full-scan mass spectrum in the orbitrap (300 –1800 m/z,

70,000 resolution) followed by 20 data-dependent MS/MS scans at 35% normalized collision energy.

The generated MS/MS spectra were searched against the Uniprot Human database (January 10, 2015; 89105 sequences) using the SEQUEST searching engine of Proteome Discoverer software (version 1.4). The search criteria were as follows: full tryptic specificity was required; one missed cleavage was allowed; carbamidomethylation (C) and TMT sixplex (K and N-terminal) were set as the fixed modifications; the oxidation (M) was set as the variable modification; precursor ion mass tolerances were set at 10 ppm for all MS acquired in an Orbitrap mass analyzer; and the fragment ion mass tolerance was set at 20 mmu for all MS2 spectra acquired. The peptide false discovery rate was calculated using Percolator provided by PD. When the q value was smaller than 1%, the peptide spectrum match was considered to be correct. False discovery was determined based on peptide spectrum match when searched against the reverse, decoy database. Peptides only assigned to a given protein group were considered as unique. The false discovery rate was also set to 0.01 for protein identifications. Relative protein quantification was performed using Proteome Discoverer software (Version 1.4) according to manufacturer's instructions on the six reporter ion intensities per peptide. Protein ratios were calculated as the median of all peptide hits belonging to a protein. Quantitative precision was expressed as protein ratio variability. Differentially expressed proteins were further confirmed by qPCR or western blotting.

Pull down assay

Cells were lysed in ice-cold 1% NP-40 lysis buffer. The lysates were incubated with anti-FLAG beads for 8h. The precipitated protein complexes were washed three times with lysis buffer without NP-40 and then suspended in protein loading buffer (contained Dithiothreitol). The samples were subjected to SDS-PAGE separation followed by LC-MS/MS analysis.

Western blotting

Cells were harvested and lysed on ice with RIPA lysis buffer. The supernatants were collected after centrifugation at 14,000 ×g for 10 min at 4°C. Protein concentrations were measured with the BCA protein assay kit. Proteins were separated in 12% SDS-PAGE gel and transferred onto a PVDF transfer membrane with electroblotting. After blocking with 5% nonfat milk for 1 h at room temperature, the membrane was incubated overnight at 4°C with primary antibody, washed with TBST buffer for 3 times, then incubated with anti-mouse or anti-rabbit secondary antibody labeled with HRP at room temperature for 1 h. The membrane was further washed with TBST buffer 3 times and developed with ECL reagents (Engreen, China). β-actin was detected with anti-β-actin antibody as an internal control. BioRad Image Lab software was used to analyze the images.

Real-time Quantitative PCR (qPCR)

The control cells and SIRT3 overexpression cells were cultured in DMEM medium. Total RNA was extracted using RNAprep pure Cell / Bacteria Kit. cDNA was

synthesized from 3 μg total RNA with the Reverse transcription kit. Quantitative real-time PCR was performed the Roche LightCycler® 480II Detection System with SYBR green incorporation according to the manufacturer's instructions and β-actin was detected as an internal control. The primers were either designed by using the Primer Premier 5 software or from Primer Bank (http://pga.mgh.harvard.edu/primerbank/). The specific PCR products were confirmed by melting curve analysis. Relative expression was analyzed using the $2^{-\Delta\Delta Ct}$ method. Primer sequences for qPCR are listed in supplemental Table S3.

Statistical Method

Statistical analysis was carried out with GraphPad Prism 5.0 software. Significant differences in the data were determined by Student's t-test. *P* values of <0.05 were considered significant.

Supplementary figures:

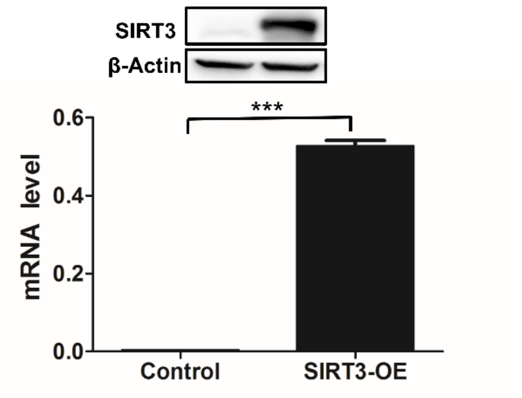

Supplementary Figure 1. qPCR and Western blotting images of SIRT3 in the control and SIRT3-OE cells.

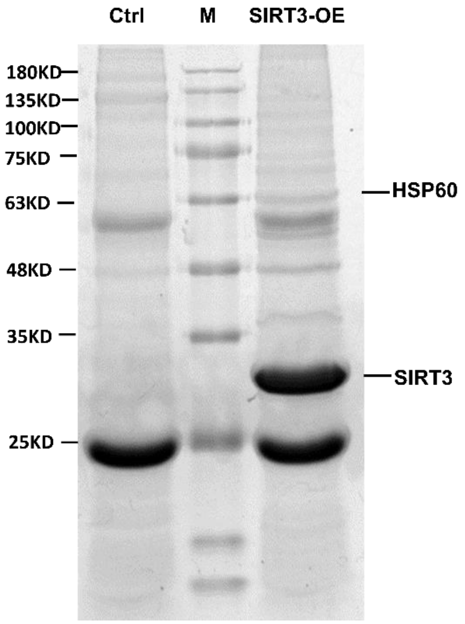

Supplementary Figure 2. 1D SDS-PAGE Separation of SIRT3-binding proteins by immunoprecipitation (IP) with an anti-FLAG antibody.

S1 Table. The list of 95 up-regulated proteins in 293T SIRT3-OE cells compared to control cells.

Accession	Description	Score	Coverage	Unique Peptides	Ratio （OE/ctrl）	MW [kDa]
P21333-2	Isoform 2 of Filamin-A	1837.8	58.7	119	1.51	279.8
P08670	Vimentin	1772.9	85.0	59	1.58	53.6
Q4VCS5	Angiomotin	1237.9	59.2	58	1.71	118.0
Q8WUM4	Programmed cell death 6-interacting protein	777.1	70.2	1	1.78	96.0
P13667	Protein disulfide-isomerase A4	742.7	67.0	59	1.53	72.9
P09936	Ubiquitin carboxyl-terminal hydrolase isozyme L1	678.9	90.1	19	2.05	24.8
Q8NC51-2	Isoform 2 of Plasminogen activator inhibitor 1 RNA-binding protein	475.7	52.7	1	3.08	44.2
P07197	Neurofilament medium polypeptide	387.0	54.4	44	1.83	102.4
P15121	Aldose reductase	375.7	72.8	18	1.52	35.8
Q9NTG7	NAD-dependent protein deacetylase sirtuin-3, mitochondrial	324.2	49.4	13	6.27	43.5
P10412	Histone H1.4	320.9	37.0	2	1.63	21.9
P58107	Epiplakin	283.2	39.5	41	1.82	555.3
O43852	Calumenin	258.8	51.1	4	1.72	37.1
Q01581	Hydroxymethylglutaryl-CoA synthase]	252.2	55.2	22	2.01	57.3
Q15942	Zyxin	251.3	48.6	20	1.60	61.2
Q9P0L0-2	Isoform 2 of Vesicle-associated membrane protein-associated protein A	244.5	38.1	1	1.65	32.6

Q9UNF1	Melanoma-associated antigen D2	218.3	45.4	30	1.54	64.9
Q6NSI4	Uncharacterized protein CXorf57	200.8	37.2	27	1.72	97.5
P07196	Neurofilament light polypeptide	184.6	34.8	19	2.09	61.5
P04792	Heat shock protein beta-1	162.7	86.8	14	1.98	22.8
J3KMY5	Epididymal secretory protein E1	144.7	52.7	10	1.51	16.2
Q16527	Cysteine and glycine-rich protein 2	123.1	63.2	12	1.51	20.9
Q15154-2	Isoform 2 of Pericentriolar material 1 protein	112.4	16.3	22	1.58	222.1
Q8N3C0	Activating signal cointegrator 1 complex subunit 3	109.9	14.4	23	1.50	251.3
Q9P0V9-3	Isoform 3 of Septin-10	98.4	24.4	6	1.64	50.0
O00762	Ubiquitin-conjugating enzyme E2 C	97.8	58.1	11	1.53	19.6
Q9H3G5	Probable serine carboxypeptidase CPVL	96.3	26.3	12	1.71	54.1
P10321	HLA class I histocompatibility antigen, Cw-7 alpha chain	95.8	31.4	1	1.65	40.6
Q16352	Alpha-internexin	95.2	14.0	2	2.07	55.4
Q8IVD9	NudC domain-containing protein 3	94.1	42.1	17	1.52	40.8
Q02818	Nucleobindin-1	91.8	50.5	19	1.60	53.8
E9PC15	Acylglycerol kinase	86.2	36.0	13	1.51	43.8
Q00534	Cyclin-dependent kinase 6	85.1	41.7	10	1.66	36.9
H0YKN4	Annexin A2	74.3	63.8	1	1.55	7.6
Q96H79	Zinc finger CCCH-type antiviral protein 1-like	73.9	57.7	12	2.33	32.9
Q9Y6H1	Coiled-coil-helix-coiled-coil-helix domain-containing protein 2	65.4	15.2	3	2.69	15.5
Q9Y6I9	Testis-expressed sequence 264 protein	63.3	27.2	8	1.65	34.2
P78559	Microtubule-associated protein 1A	60.9	7.5	14	1.51	305.3
P84101-4	Isoform 4 of Small EDRK-rich factor 2	54.0	42.2	4	2.28	5.2
Q9NS86	LanC-like protein 2	51.7	13.8	5	1.53	50.8
Q99442	Translocation protein SEC62	50.9	11.8	6	1.72	45.8
Q9P2B2	Prostaglandin F2 receptor negative regulator	49.6	16.7	11	1.59	98.5
Q6P1Q9	Methyltransferase-like protein 2B	44.4	37.0	2	1.59	43.4
P45877	Peptidyl-prolyl cis-trans isomerase C	43.4	17.5	3	1.51	22.7
A8MWK3	Cadherin-2	41.2	11.7	6	1.59	97.0
Q9BT09	Protein canopy homolog 3	39.0	35.6	8	1.65	30.7
Q8N556	Actin filament-associated protein 1	36.6	5.2	4	2.51	80.7
E7ET33	Inter-alpha-trypsin inhibitor heavy chain H3	36.5	4.4	3	2.66	78.1
H0YKX3	PCNA-associated factor	36.2	58.3	4	1.75	9.3
P09543-2	Isoform CNPI of 2',3'-cyclic-nucleotide 3'-phosphodiesterase	35.8	23.2	8	1.66	45.1
P47974	Zinc finger protein 36, C3H1 type-like 2	33.6	17.8	7	1.61	51.0
Q8N0U8	Vitamin K epoxide reductase complex subunit 1-like protein 1	31.6	5.7	1	1.60	19.8
Q9BXB5-2	Isoform 2 of Oxysterol-binding protein-related protein 10	29.8	14.9	5	1.51	77.2
O15231-9	Isoform 9 of Zinc finger protein 185	29.0	25.7	5	2.23	34.9

H0YA55	Serum albumin (Fragment)	27.4	7.7	4	1.54	51.5
A6NFI3	Zinc finger protein 316	27.2	2.5	2	1.57	108.4
E9PM62	Protein wntless homolog (Fragment)	26.3	18.5	6	1.65	26.2
A8MY76	Nucleolar protein 9	25.9	19.6	1	1.51	58.1
Q9Y3Q3	Transmembrane emp24 domain-containing protein 3	24.8	23.0	4	1.67	24.8
Q9UJC5	SH3 domain-binding glutamic acid-rich-like protein 2	24.2	30.8	3	1.63	12.3
P80723	Brain acid soluble protein 1	24.2	52.4	6	2.42	22.7
Q5VZR0	Golgi-associated plant pathogenesis-related protein 1	22.0	37.5	3	1.57	14.2
Q9Y605	MORF4 family-associated protein 1	20.1	37.8	3	1.76	14.6
Q86Y46-2	Isoform 2 of Keratin, type II cytoskeletal 73	18.3	5.5	1	1.54	42.0
P02765	Alpha-2-HS-glycoprotein	18.1	6.8	4	1.75	39.3
Q9Y385	Ubiquitin-conjugating enzyme E2 J1	16.9	15.7	4	1.60	35.2
P04920-2	Isoform B1 of Anion exchange protein 2	16.8	5.2	5	1.98	135.5
Q9UHF1	Epidermal growth factor-like protein 7	16.7	20.2	4	1.55	29.6
P50150	Guanine nucleotide-binding protein G(I)/G(S)/G(O) subunit gamma-4	14.7	32.0	2	1.53	8.4
O75503	Ceroid-lipofuscinosis neuronal protein 5	14.3	5.0	2	1.79	41.5
F5GXP4	ADP-ribosylation factor-like protein 6-interacting protein 1	14.2	13.2	2	1.53	20.2
P01034	Cystatin-C	14.2	19.2	2	1.78	15.8
Q14999	Cullin-7	14.1	1.1	2	2.30	191.0
P09629	Homeobox protein Hox-B7	13.7	11.5	1	1.79	24.0
Q16626	Male-enhanced antigen 1	13.6	7.0	1	2.17	19.9
Q2T9J9	TTC7A protein	13.4	0.7	1	2.75	98.9
H0YGH6	Alpha-2-macroglobulin (Fragment)	13.2	13.0	2	2.57	21.9
P20742	Pregnancy zone protein	13.1	1.4	1	1.51	163.8
Q9Y4H2	Insulin receptor substrate 2	12.7	2.2	3	1.68	137.2
A6NGB9	WAS/WASL-interacting protein family member 3	12.0	10.6	3	1.63	49.4
Q9HBK9-2	Isoform 2 of Arsenite methyltransferase	11.9	17.2	4	1.74	31.1
B4DL05	Protein SHQ1 homolog	11.5	4.7	3	1.72	61.5
Q8N0T1	Uncharacterized protein C8orf59	9.1	32.0	4	1.71	11.4
P39060-2	Isoform 3 of Collagen alpha-1(XVIII) chain	8.8	3.1	2	1.96	135.7
P18065	Insulin-like growth factor-binding protein 2	7.6	7.7	2	1.86	34.8
Q5VUM1	UPF0369 protein C6orf57	6.9	36.1	2	1.89	12.2
B4DJN5	Protein kinase C eta type	6.8	6.5	1	1.67	59.5
Q14118	Dystroglycan	6.8	1.7	1	1.55	97.4
Q96S97	Myeloid-associated differentiation marker	6.2	1.9	1	1.53	35.3
B4E1B9	Transmembrane protein 2	6.0	7.5	1	1.97	42.4
Q8WVD3	E3 ubiquitin-protein ligase RNF138	5.8	11.8	3	1.73	28.2

Q9NUN5-4	Isoform 4 of Probable lysosomal cobalamin transporter	5.5	9.6	1	1.51	21.4
P36955	Pigment epithelium-derived factor	5.4	6.5	2	1.53	46.3
P98172	Ephrin-B1	5.2	5.2	1	1.51	38.0
H0YFA4	Cysteine-rich protein 2 (Fragment)	5.1	8.3	1	1.77	20.7

S2 table. The list of 93 down-regulated proteins in 293T SIRT3-OE cells compared to control cells.

Accession	Description	Score	Coverage	Unique Peptides	Ratio (OE/ctrl)_	MW [kDa]
P06733-2	Isoform MBP-1 of Alpha-enolase	3390.6	84.5	1	0.55	36.9
Q05639	Elongation factor 1-alpha 2	1741.4	77.3	16	0.48	50.4
P17066	Heat shock 70 kDa protein 6	1122.3	24.1	1	0.60	71.0
P00918	Carbonic anhydrase 2	746.3	72.3	25	0.57	29.2
P26038	Moesin	704.6	81.8	48	0.57	67.8
F8VZX2	Poly(rC)-binding protein 2	613.5	80.7	1	0.61	33.8
Q9UHD1	Cysteine and histidine-rich domain-containing protein 1	542.2	81.3	28	0.65	37.5
P19367-4	Isoform 4 of Hexokinase-1	468.8	50.4	46	0.66	101.0
P00505	Aspartate aminotransferase	465.9	69.3	32	0.65	47.5
P06493	Cyclin-dependent kinase 1	379.2	69.4	18	0.66	34.1
P30084	Enoyl-CoA hydratase	314.6	75.2	23	0.56	31.4
K7ELW5	Polypyrimidine tract-binding protein 1	306.8	66.1	1	0.49	24.3
Q8IUE6	Histone H2A type 2-B	293.6	40.8	1	0.64	14.0
Q7L0Y3	Mitochondrial ribonuclease P protein 1	285.0	72.2	28	0.64	47.3
P11802	Cyclin-dependent kinase 4	275.2	64.0	16	0.56	33.7
J3KP15	Serine/arginine-rich-splicing factor 2 (Fragment)	265.1	45.9	5	0.56	15.4
Q9H2U2	Inorganic pyrophosphatase 2	252.0	69.2	24	0.62	37.9
G5E9W7	28S ribosomal protein S22	239.1	51.7	15	0.65	36.8
P30405	Peptidyl-prolyl cis-trans isomerase F	223.2	79.2	16	0.54	22.0
P21912	Succinate dehydrogenase [ubiquinone] iron-sulfur subunit	209.7	59.6	17	0.58	31.6
P49748-2	Isoform 2 of Very long-chain specific acyl-CoA dehydrogenase	194.6	47.6	25	0.63	68.0
Q8N684-2	Isoform 2 of Cleavage and polyadenylation specificity factor subunit 7	153.8	40.3	17	0.59	51.1
Q9P0M6	Core histone macro-H2A.2	140.4	52.4	14	0.60	40.0
Q7Z7K6-3	Isoform 3 of Centromere protein V	132.4	69.5	13	0.62	29.7
Q06265	Exosome complex component RRP45	129.4	36.7	11	0.65	48.9
P42126	Enoyl-CoA delta isomerase 1	121.3	38.1	9	0.53	32.8
Q9BYD6	39S ribosomal protein L1	114.6	52.6	17	0.66	36.9
Q00059	Transcription factor A	111.3	52.0	17	0.65	29.1
Q05193-3	Isoform 3 of Dynamin-1	110.3	26.4	10	0.62	96.0

P42771	Cyclin-dependent kinase inhibitor 2A, isoforms 1/2/3	109.0	60.3	7	0.50	16.5
Q71DI3	Histone H3.2	105.0	55.9	1	0.57	15.4
Q14019	Coactosin-like protein	102.2	90.1	5	0.65	15.9
Q9ULX3	RNA-binding protein NOB1	99.5	30.1	13	0.61	46.6
Q13257	Mitotic spindle assembly checkpoint protein MAD2A	91.6	35.1	8	0.62	23.5
Q6NUK1-2	Isoform 2 of Calcium-binding mitochondrial carrier protein SCaMC-1	84.9	28.0	10	0.67	51.3
Q3SY69	Mitochondrial 10-formyltetrahydrofolate dehydrogenase	78.2	18.5	14	0.58	101.7
O15479	Melanoma-associated antigen B2	76.4	32.9	11	0.28	35.3
Q9UDR5	Alpha-aminoadipic semialdehyde synthase	73.0	21.8	14	0.59	102.1
Q9Y305-3	Isoform 3 of Acyl-coenzyme A thioesterase 9	70.2	43.0	13	0.67	43.2
P53007	Tricarboxylate transport protein	66.7	46.6	11	0.65	34.0
B7Z6B8	2,4-dienoyl-CoA reductase	62.2	51.2	13	0.61	35.0
A3KMH1-2	Isoform 2 of von Willebrand factor A domain-containing protein 8	56.0	21.0	1	0.67	116.9
Q9NXV6	CDKN2A-interacting protein	55.4	24.8	10	0.66	61.1
Q9UII2	ATPase inhibitor	55.1	43.4	7	0.58	12.2
P35612	Beta-adducin	53.0	20.4	11	0.63	80.8
D6RBR7	Zinc finger protein 330 (Fragment)	50.6	31.4	7	0.57	25.8
D6R9Z7	Cytochrome c oxidase subunit 7C	49.6	42.9	4	0.65	6.4
Q9UFN0	Protein NipSnap homolog 3A	48.7	47.8	8	0.64	28.4
P56945-4	Isoform 4 of Breast cancer anti-estrogen resistance protein 1	47.6	7.5	5	0.61	77.7
Q9BQ69	O-acetyl-ADP-ribose deacetylase MACROD1	47.5	43.7	10	0.65	35.5
Q5SVL2	Caspase-7 (Fragment)	47.3	38.3	7	0.56	24.8
P04264	Keratin, type II cytoskeletal 1	45.7	9.2	5	0.35	66.0
Q9Y692-2	Isoform 2 of Glucocorticoid modulatory element-binding protein 1	42.5	13.9	5	0.65	61.3
Q9NX01	Thioredoxin-like protein 4B	39.5	31.5	4	0.57	17.0
C9JA36	Sodium/potassium-transporting ATPase subunit beta-3 (Fragment)	37.6	75.9	1	0.54	15.0
O75616	GTPase Era	35.6	28.2	10	0.62	48.3
B7Z5N5	Mothers against decapentaplegic homolog 2	35.1	17.6	1	0.67	48.6
P14406	Cytochrome c oxidase subunit 7A2	34.9	30.1	3	0.64	9.4
Q14195-2	Isoform LCRMP-4 of Dihydropyrimidinase-related protein 3	34.5	15.9	4	0.45	73.9
C9JYQ9	60S ribosomal protein L22-like 1	31.8	46.3	3	0.47	14.5
Q14331	Protein FRG1	28.7	18.6	7	0.62	29.2
P56181	NADH dehydrogenase [ubiquinone] flavoprotein 3	28.2	26.9	1	0.59	11.9
Q96E29-3	Isoform 3 of mTERF domain-containing protein 1	27.6	18.9	5	0.63	34.3
B9A057	Cytochrome c oxidase assembly factor	24.7	11.0	1	0.63	8.1

Q53S33	BolA-like protein 3	24.6	41.1	6	0.56	12.1
Q9BQ48	39S ribosomal protein L34	24.3	10.9	1	0.62	10.2
Q9H008	Phospholysine phosphohistidine inorganic pyrophosphate phosphatase	23.6	17.4	4	0.61	29.1
P31327-2	Isoform 2 of Carbamoyl-phosphate synthase [ammonia]	22.8	5.6	2	0.52	116.0
P32321	Deoxycytidylate deaminase	21.4	18.5	2	0.55	20.0
Q16773-2	Isoform 2 of Kynurenine--oxoglutarate transaminase 1	20.4	7.8	2	0.64	42.5
Q9NPG3-2	Isoform 2 of Ubinuclein-1	19.8	5.4	4	0.64	118.5
Q96A00	Protein phosphatase 1 regulatory subunit 14A	18.7	23.8	4	0.53	16.7
Q8IWL3	Iron-sulfur cluster co-chaperone protein HscB	18.2	26.4	5	0.60	27.4
F5H7F6	Microsomal glutathione S-transferase 1 (Fragment)	16.4	50.7	5	0.48	8.9
I3L0Y6	NmrA-like family domain-containing protein 1 (Fragment)	16.4	17.6	3	0.42	20.8
P78549-3	Isoform 3 of Endonuclease III-like protein 1	16.2	8.8	2	0.62	32.8
P56377	AP-1 complex subunit sigma-2	16.1	22.9	3	0.64	18.6
P0CG34	Thymosin beta-15A	15.7	55.6	4	0.56	5.2
K7ESB0	Centromere protein S (Fragment)	14.7	30.3	2	0.45	8.9
H3BU49	ADP-ribosylation factor-like protein 2-binding protein	10.8	22.0	2	0.67	13.8
O60662	Kelch-like protein 41	10.4	10.1	2	0.57	68.0
F5GZY7	Gamma-aminobutyric acid receptor-associated protein-like 1 (Fragment)	10.0	34.3	1	0.64	8.6
P13284	Gamma-interferon-inducible lysosomal thiol reductase	9.9	20.0	2	0.54	27.9
G3XAJ6	Raft-linking protein, isoform CRA_c	9.4	5.0	2	0.67	58.7
Q9HC07	Transmembrane protein 165	8.4	10.2	2	0.63	34.9
J3QR01	Centromere protein X (Fragment)	8.3	41.3	2	0.55	8.3
Q9H477-2	Isoform 2 of Ribokinase	7.5	7.0	2	0.62	30.3
P12074	Cytochrome c oxidase subunit 6A1	7.1	26.6	1	0.57	12.1
Q9BQE5	Apolipoprotein L2	6.9	8.6	3	0.37	37.1
Q02487-2	Isoform 2B of Desmocollin-2	6.8	2.0	2	0.65	93.7
Q9Y619	Mitochondrial ornithine transporter 1	6.6	6.6	2	0.67	32.7
K7EIN2	Protein syndesmos (Fragment)	6.0	26.8	4	0.66	21.9
O95359-2	Isoform 2 of Transforming acidic coiled-coil-containing protein 2	5.6	2.6	1	0.67	64.1
J3KT99	39S ribosomal protein L45	5.4	45.7	1	0.58	4.2

S3 table. Primers used in this work

Primer name	Primer sequence
CDK4-F	ATGGCTACCTCTCGATATGAGC
CDK4-R	CATTGGGGACTCTCACACTCT
CDK1-F	AAACTACAGGTCAAGTGGTAGCC
CDK1-R	TCCTGCATAAGCACATCCTGA
SDHB-F	ACAGCTCCCCGTATCAAGAAA
SDHB-R	GCATGATCTTCGGAAGGTCAA
COA7-F	CTGCCAAGGTGTTGAAGTTTAAC
COA7-R	AGGCTTCTCACACGCCATC
MRPL17-F	CCGCGTATTTCGCCGTATG
MRPL17-R	CATGGTGCCTCGATGCGTT
MRPS9-F	CAGTTCATTCGGCTGCTAGAA
MRPS9-R	AGGCCATTCCTTGCTCATCAT
MRPS6-F	GGCCAGAGACTGCTGCTAC
MRPS6-R	GCGGTGGGTGCATAAAAATCC
MRPL49-F	GCTACCATGTTCCGGGCTAC
MRPL49-R	CAGGCGCTCCACAAACTGATA
MRPS21-F	ACAGAATCCTCACTATGGATGGG
MRPS21-R	AAGAAGTTGATCTTGCGAGCC
MRPL1-F	GGAAGGCGAACCTGAGGATG
MRPL1-R	TGCCATATCCAGTGTCAAATCAA
MRPS22-F	ACCGCTACCTTGCTCTTTCG
MRPS22-R	TGGCTTCAGTTCTTGTATAGCTG
MRPL45-F	TCCAGAAAAATCGGACCGTTC
MRPL45-R	GTTCTCTCTATCAGTCCCTCCTT
MRPL34-F	TGCCCCGCGGATATAGACT
MRPL34-R	GATCCAGCCAAGACAGCCATA
SIRT3-F	CCCCAAGCCCTTTTTCACTTT
SIRT3-R	CGACACTCTCTCAAGCCCA
